# Gynaecological diagnosis by ultrasound and the measurement of urinary sex steroid hormones in female orangutans

**DOI:** 10.1002/vms3.237

**Published:** 2020-01-25

**Authors:** Kodzue Kinoshita, Tomoyuki Nakamura, Koichi Kimura, Mika Shimizu, Noko Kuze, Yasuhiko Ozaki

**Affiliations:** ^1^ Wildlife Research Center Kyoto University Kyoto Japan; ^2^ Chiba Zoological Park Chiba Japan; ^3^ Nagoya Higashiyama Zoo and Botanical Gardens Aichi Japan; ^4^ Tama Zoological Park Tokyo Japan; ^5^ Japan Society for the Promotion of Science Tokyo Japan; ^6^ The National Museum of Nature and Science Ibaraki Japan; ^7^ Department of Obstetrics and Gynecology The Education and Research Center for Advanced Medicine Graduate School of Medical Sciences Nagoya City University Aichi Japan

**Keywords:** endometrium, oestrogen, ovary, ovulation, reproductive organ

## Abstract

Gynaecological diagnoses were carried in three adult female orangutans (*Pongo* spp.) using ultrasound, and their estrous states were estimated by measuring urinary sex steroid hormone concentrations using enzyme immunoassay. Ultrasound diagnosis revealed that the endometrial thickness and follicle size were correlated with the oestrogen‐3‐glucuronide concentrations in the follicular phase. In addition, administration of the ovulation inducer human chorionic gonadotropin (hCG) had the strongest effect on the pregnanediol‐3‐glucuronide (PdG) concentration when the follicle size was 22.3 mm, suggesting that the follicle reaches this size before ovulation. The similarity between this and the maximum ovarian follicle size in humans (approximately 20 mm) indicates that the ancestral reproductive characteristics may have been retained in these species.

## Graphical abstract

In orangutans, endometrium thickness and follicle size were correlated with estrogen concentration. And follicle may increase to around 22.3 mm prior to ovulation. Orangutans and humans have similar reproductive physiology.

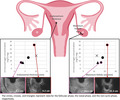

## 1 INTRODUCTION

The orangutan typically has an older age at first reproduction (15 years old), a longer interbirth interval (7.6 years) (van Noordwijk et al., 2018) and a longer generation time (239–254 days) (Kinoshita et al., 2017) than African apes (Wich et al., 2004). This species also has a lower breeding efficiency than other apes, with a relatively low rate of reproduction, despite the females being polyestrous and thus having a comparatively higher number of breeding opportunities. There has been only one example of successful artificial insemination (AI) to date (Forde, 2014). Therefore, information on the reproductive physiology of this species is required to improve captive breeding success and thus conserve genetic variability.

The reproductive physiology of orangutan has previously been examined by measuring the concentrations of several sex hormones in urine collected from captive individuals, particularly females (Aramaki et al., 2010; Kinoshita et al., 2017). However, to the best of our knowledge, the relationship between changes in hormone concentrations and the reproductive tract in cyclic female orangutans has not yet been investigated. Therefore, in the present study, we undertook gynaecological diagnostic tests to gather information on the reproductive tracts and measured hormonal concentrations in female orangutans.

## 2 MATERIALS AND METHODS

The morphology of the reproductive organs (dimensions of longitudinal and transverse sections of the uterus, uterine lumen diameter, cervix length, endometrial thickness and follicle size) was observed and measured in three adult females by ultrasound diagnosis under anaesthesia. The three females evaluated were: F1 (ID: 0101 in the Great Ape Information Network; http://www.shigen.nig.ac.jp/gain/; 24 years old at the start of the present study, Bornean orangutan), F2 (ID: 0082; 30 years old, Sumatran orangutan) and F3 (ID:0011; 50 years old, Bornean orangutan). These individuals were housed in separate indoor sleeping rooms and an outdoor exhibition area and were fed fresh fruits and vegetables and provided with water ad libitum. In addition, to confirm whether the females were in the follicular phase or the luteal phase, urinary estrone ‐3‐glucuronide (E_1_G) and pregnanediol‐3‐glucuronide (PdG) concentrations were measured by enzyme immunoassay as previously described (Kinoshita et al., 2017) and were corrected using the creatinine concentration. In F1, ultrasound diagnoses were performed for AI on days when E_1_G levels were increased, as predicted from the hormonal results based on Aramaki et al. (2010), and an ovulation inducer [human chorionic gonadotropin (hCG): 10,000 IU] was then administered. In F2 and F3, the diagnoses were performed on the same day as each individual received an anaesthetic for medical attendance (F2, vaginal prolapse; F3, empyema). All animal manipulations were performed according to the Word Association of Zoos and Aquariums Ethical Guidelines for the Conduct of Research on Animals by Zoos and Aquariums, and ultrasound diagnoses and AI under anaesthesia were approved by the Animal Welfare and Animal Care Committee of the Primate Research Institute (#2015‐139–04) of Kyoto University, Japan, based on the Guidelines for Care and Use of Non‐human Primates (Version 3, issued in 2010).

## 3 RESULTS

**Figure 1:**
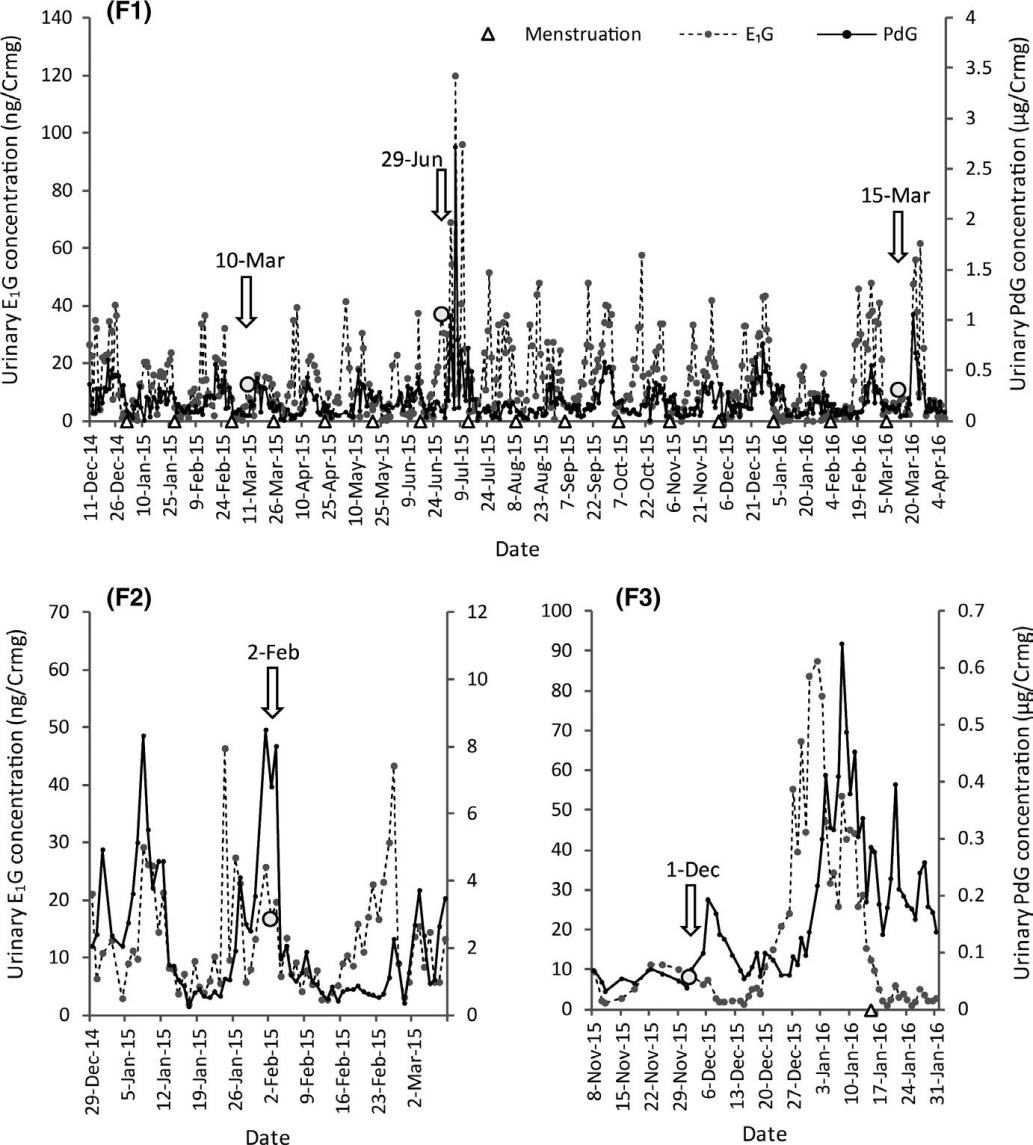
Urinary sex steroid hormone concentrations in three adult female orangutans (*Pongo* spp.). Each triangle represents the first day of menstruation (Note: in F2, it was difficult to determine whether the female was menstruating or simply had a cut on the vagina due to vaginal prolapse, so menstruation was not recorded). The arrows and white circles in association with E_1_G indicate the days when the reproductive organs were measured by ultrasound diagnosis. E_1_G, estrone‐3‐glucuronide; PdG, pregnandiol‐3‐glucuronide

**Figure 2:**
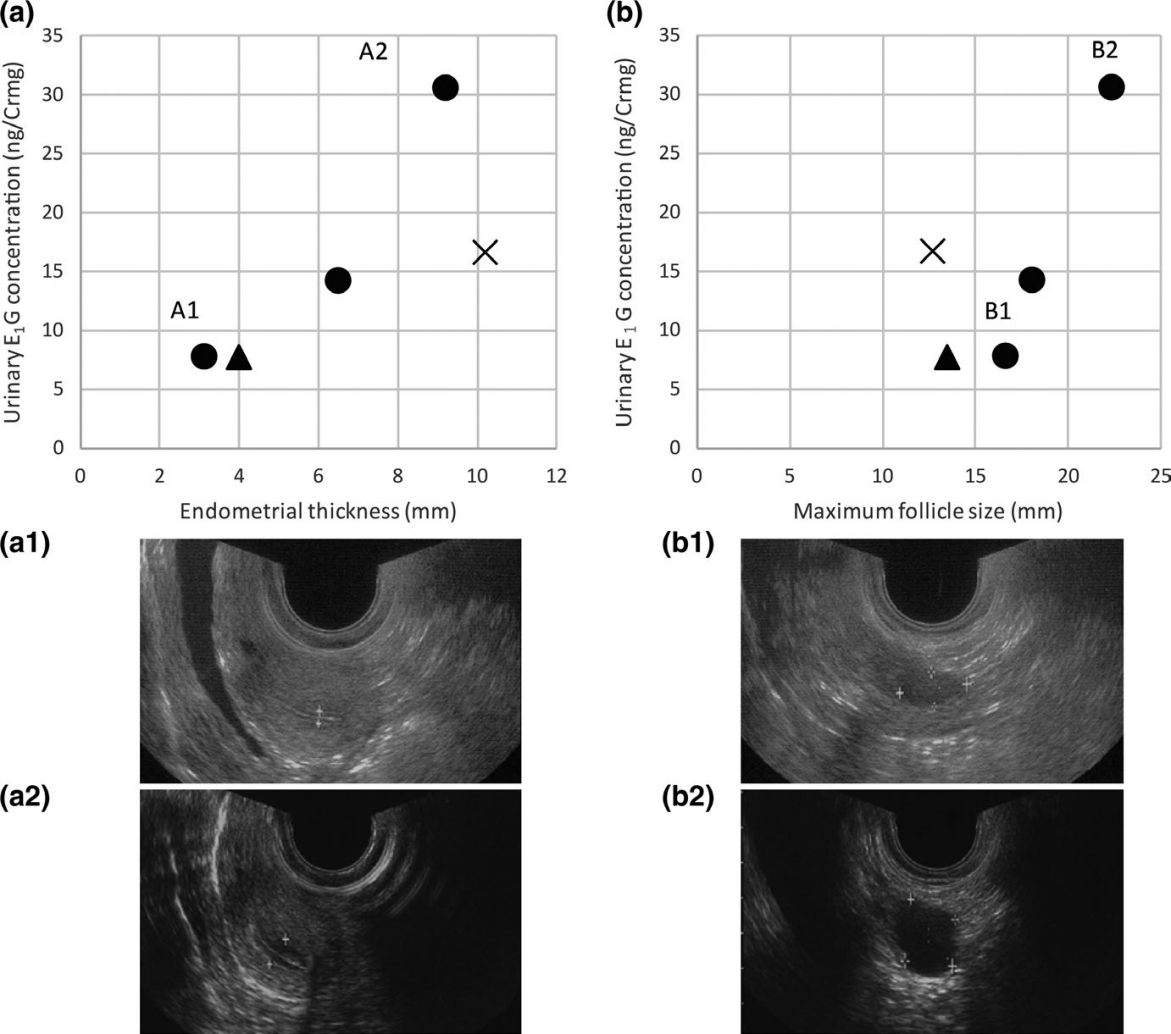
Relationships between the urinary estrone‐3‐glucuronide (E_1_G) concentration, the endometrial thickness (a), and the maximum follicle size (b) in three adult female orangutans (*Pongo* spp.). The circles, crosses and triangles represent data for the follicular phase of F1, the luteal phase of F2 and the non‐cyclic phase of F3, respectively. The ultrasonographic images were taken during the follicular phase in F1 on days when the E_1_G concentration was low (A1 and B1; March 15, 2016) and high (A2 and B2; June 29, 2015)

Ultrasound diagnoses were conducted in the follicular phase (*n* = 3; F1), the luteal phase (*n* = 1; F2) and the non‐cyclic phase (*n* = 1; F3) (Figure 1). The mean length of the menstrual cycle in F1, where monitoring was undertaken over a long period of time, was 28.9 ± 2.0 days [mean ± standard deviation (*SD*)], with the follicular and luteal phases lasting 12.9 ± 1.5 days and 16.0 ± 1.1 days, respectively (*n* = 16). The results of the ultrasound diagnoses and urinary E_1_G concentrations are shown in Table 1. The ultrasound diagnoses revealed that the endometrial thickness (range: 4.0–9.2 mm) and the follicle size (range: 13.5–22.3 mm) were correlated with the E_1_G concentration (range: 7.67–30.63 ng/Crmg) in the follicular phase and were smallest in the non‐cyclic phase (Figure 2). For F1, a much higher PdG concentration was obtained after the administration of hCG on June 29, 2015, when the follicle was 22.3 mm in diameter (Figure 1).

**Table 1:** Reproductive organ measurements by ultrasound diagnosis and urinary estrone‐3‐glucuronide (E_1_G) concentrations in three adult female orangutans (*Pongo* spp.)

Individual	Date	Uterus		Uterine lumen	Cervix length	Endometrial thickness	Right follicle		Left follicle		Maximum follicle size	Urinary E_1_G concentration
		Longitudinal(mm)	Transverse(mm)	(mm)	(mm)	(mm)	Number	(mm)	Number	(mm)	(mm)	(ng/Crmg)
F1	Mar−10–15	27.9 × >60	31.3 × 32.9	32	26	6.5	0	–	1	16.7 × 18.0	18	14.3
	Jun−29–15	–	–	–	–	9.2	1	22.3 × 18.5	1	17.2 × 12.0	22.3	30.6
	Mar−15–16	30.4 × 56.6	–	–	–	3.1	2	5.2, 6.7	2	16.6 × 8.4, 8.7 × 3.8	16.6	7.9
F2	Feb−2–15	45 × 79.7	–	–	–	10.2	2	12.7	1	12.5	12.7	16.6
								12.7				
F3	Dec−1–15	31.5 × 56	–	–	–	4	0	–	3	10.1	13.5	7.7
		38.8 × 56.9								8		
										12.9 × 13.5		

## 4 DISCUSSION

Asa et al. (1995) demonstrated that an apparently mature follicle was 20 mm in diameter in an orangutan that had been administrated follicle stimulating hormone and luteinizing hormone. Similarly, the maximum ovarian follicle size is approximately 23 mm for humans (Kerin et al., 1981) and > 20 mm for gorillas (*Gorilla gorilla gorilla*) (Huntress et al., 1989), whereas it is much smaller (approximately 10 mm) for chimpanzees (*Pan troglodytes*) (Graham, Keeling, Chapman, Cummins, & Haynie, 1973). This study also suggested that the follicles may increase to approximately 22.3 mm before ovulation because the hCG had the greatest effect at that time.

Bakos, Lundkvist, Wide, and Bergh (1994) reported that mature follicles in humans had a mean diameter of 21.4 mm at ovulation, whereas the endometrium had a mean thickness of 12.8 mm (range: 10.0–15.9 mm), which was larger than observed in the present study. Although only one sample was observed in the luteal phase, the endometrial thickness at this stage was the widest in all samples. Therefore, the accumulation of more data in the luteal phase may yield a wider size than is observed in humans.

The mean length of the menstrual cycle in this study was quite similar to humans [mean length of menstruation cycle: 28 days (range: 25–30 days); mean length of follicular phase: 14.4 days (range: 12–17 days); mean length of luteal phase: 13.5 days (range: 12–16 days); (Bakos et al., 1994)]. By contrast, the menstrual cycle is known to be longer in the other great apes: 28–38 days for gorillas (Dahl, Czekala, Lim, & Hsueh, 1987) and 31–46 days for chimpanzees (Young & Yerkes, 1943). Furthermore, both orangutans and humans are intermediate between chimpanzees and gorillas in terms of the male reproductive traits (e.g., testes size, degree of semen coagulation and sperm midpiece volume) (Carnahan & Jensen‐Seaman, 2008).

Together, these findings indicate that although the orangutan lineage is most distant from the human lineage among the great apes, these species have more similar reproductive physiologies than the other great apes. Thus, the characteristics of the ancestral species may have been well conserved in the orangutan and human, while the chimpanzee and gorilla may have evolved specialized characteristics. The gynaecological diagnosis by ultrasound and measurement of urinary sex steroid hormone concentrations were thought to be useful for reproductive medicine and to plan breeding protocols, including AI in orangutans like humans.
